# Efficacy of Tong-Xie-Yao-Fang granule and its impact on whole transcriptome profiling in diarrhea-predominant irritable bowel syndrome patients: study protocol for a randomized controlled trial

**DOI:** 10.1186/s13063-020-04833-x

**Published:** 2020-11-03

**Authors:** Yan Wang, Yong-quan Huang, Shui-lian Zhu, Chang-rong Zhang, Xin-lin Chen, Qiu-ke Hou, Feng-bin Liu

**Affiliations:** 1 Department of Gastroenterology, The First Affiliated Hospital of Guangzhou University of Chinese Medicine, Guangzhou, China; 2 Department of Orthopedics, The Second Affiliated Hospital of Guangzhou University of Chinese Medicine, Guangzhou, China; 3 Department of Preventive Medicine and Health Statistics, Guangzhou University of Chinese Medicine, Guangzhou, China; 4 School of Chinese Medicine, Hong Kong Baptist University, Hong Kong, China

**Keywords:** Diarrhea-predominant irritable bowel syndrome, Whole transcriptome profiling, Tong-Xie-Yao-Fang granule, Randomized controlled trial, Traditional Chinese medicine

## Abstract

**Background:**

Irritable bowel syndrome (IBS) is one kind of common functional bowel disease with obscure pathogenesis, and exploration about whole transcriptome profiling in IBS-D is still negligible. Conventional medications have limited effects, which makes focus shifted to traditional Chinese medicine (TCM). Tong-Xie-Yao-Fang, as a classic herbal formula in TCM, is pretty effective and safe for the treatment of diarrhea-predominant irritable bowel syndrome (IBS-D), but the underlying therapeutic mechanism remains unknown. We aim to verify the efficacy and safety of TXYF granule (the formula particles mixed together) in IBS-D and elucidate the gene-level mechanism of IBS-D and therapeutic targets of TXYF granule based on whole transcriptome analysis.

**Methods/design:**

This is a randomized, double-blind, and placebo-controlled clinical trial consisting of 2 weeks of run-in period, 12 weeks of treatment period, and 8 weeks of follow-up period. We will enroll 120 participants with IBS-D, who will be randomly assigned to the TXYF granule group and the placebo group, and recruit additional 10 healthy individuals as controls for mechanistic outcome. The two groups respectively take TXYF granule or placebo orally for treatment. The primary outcome is the response rate of IBS-Symptom Severity Score (IBS-SSS). The secondary outcomes include adequate relief (AR), IBS-Quality of Life Questionnaire (IBS-QOL), and long-term efficacy. Mechanistic outcome is the whole transcriptome profiling of the intestinal mucosae from IBS participants before and after the treatment and healthy individuals.

**Discussion:**

This trial will prove the effectiveness and safety of TXYF granule with high-quality evidence and provide a penetrating and comprehensive perspective on the molecular mechanism of IBS-D by whole transcriptome analysis, which makes us pinpoint specific biomarkers of IBS-D and therapeutic targets of TXYF.

**Trial registration:**

Chinese Clinical Trial Registry ChiCTR-IOR-1900021785. Registered on 9 March 2019

## Background

As a common functional bowel disease, irritable bowel syndrome (IBS) is mainly characterized by intermittent abdominal pain, disturbed intestinal motility, and altered bowel habits, which can be elaborately categorized into four subtypes [1]. Thereinto, diarrhea-predominant irritable bowel syndrome (IBS-D) defined as loose or watery stools (precisely type 6 or type 7 of Bristol stool form scale) appearing more than 1/4 of bowel movements (BM) and less than 1/4 of BM with type 1 or type 2 of Bristol stool form scale definitely is the most common subtype of IBS [2]. From the perspective of epidemiology, this nuisance ailment affects 10 to 15% of the population and females are predisposed to this disease [3]. IBS is a multifactorial disease, and many studies have demonstrated that visceral hypersensitivity [4], gut barrier dysfunction [5], aberrant microbiota-brain-gut interaction [6, 7], and intestinal motility abnormality [8] are the pivotal participators in the pathogenesis. Deserve to be mentioned, further studies at the gene level are imperative because they are conducive to decipher the aforementioned pathogenesis. Conventional medications for IBS-D, such as antidiarrheal, antispasmodic, antibiotics, and probiotics, often have limited effects and repeated treatment brings about tremendous socioeconomic pressure so that the therapeutic focus has naturally shifted to traditional Chinese medicine (TCM).

Tong-Xie-Yao-Fang (TXYF), a classic prescription in Chinese herbal medicine, consisting of four core herbs (*Rhizoma Atractylodis Macrocephalae*, *Paeoniae Radix Alba*, *Citri Reticulatae Pericarpium*, and *Saposhnikoviae Radix*), has been specially applied for mitigating abdominal pain accompanied with diarrhea in clinics for hundreds of years. TXYF accords with one kind of major pathogenesis known as liver depression and spleen deficiency of IBS-D in TCM theory. *Rhizoma Atractylodis Macrocephalae* and *Citri Reticulatae Pericarpium* strengthen the spleen to eliminate dampness while *Paeoniae Radix Alba* and *Saposhnikoviae Radix* soothe the liver to regulate qi. These four herbs contained in TXYF by orchestrating the function of the spleen and liver to relieve symptoms of IBS-D. A recent study has proved that TXYF is efficacious in the relief of abdominal pain and bloating in IBS-D patients with only sporadic adverse events [9]. But there is a scrap of contradictory evidence that TXYF has little impact on the amelioration of symptoms of IBS-D [10]. Meanwhile, it has been demonstrated in IBS-D rat models that TXYF can regulate the level of 5-HT and SP with respect to visceral hypersensitivity and TXYF can modulate the intestinal flora by downregulating the ratio of *Firmicutes* to *Bacteroidetes* and the fecal abundance of *Clostridium* [11, 12]. Moreover, TXYF has been proved to ameliorate intestinal permeability by inhibiting inflammation-related NF-κB and notch pathways [13]. However, heterogeneity about the constitution and the dose of TXYF exists in previous studies. TXYF therapeutic targets and molecular mechanisms directing IBS-D have not been profoundly illuminated.

About 75% of human genes can be transcribed, and transcriptome landscape is the direct reflection of encoded genetic information [14]. Transcriptome profiling is able to comprehensively unveil the pathogenesis of IBS-D and the targets of traditional Chinese medicine by analyzing the interaction networks of differentially expressed protein-coding RNAs and noncoding RNAs (ncRNAs). Two pilot studies show that by using RNA-seq and RT-PCR, there is a discrepancy in rectosigmoid/small bowel mucosal mRNA expression between IBS-D patients and controls, mainly involving neurotransmitters, intestinal immunity, barrier function, ion transport, and so on [15, 16]. In the WAS-induced IBS rat model, the transcriptome profile demonstrates that the majority of differentially expressed genes relate to notch signaling and focal adhesion by employing mRNA microarray analysis [17].

Along with the blooming of the RNA sequencing technology, noncoding RNAs, previously thought to be “transcriptional noise,” actually have been proven crucial to transcriptional and post-transcriptional events of gene expression and genome maintenance [18]. More and more studies have given insights into the indispensable roles of ncRNAs played in the cellular process [19], tumorigenesis [20], inflammatory regulation [21], and so on. Our previous studies also have reported that microRNA-144 and microRNA-200a are involved in intestinal permeability and visceral hyperalgesia separately by binding to specific target mRNA in IBS-D rats [22, 23]. However, exploration about ncRNAs for IBS-D is still negligible. Few studies shed light on the function of long noncoding RNA and circular RNA in the pathogenesis of IBS-D. Nor do researchers probe the whole transcriptome profiling for targets or pathways of TCM in IBS-D. In the current trial, we attempt to verify the efficacy and safety of TXYF granule (the formula particles mixed together) for the treatment of IBS-D, which syndrome differentiated as liver depression and spleen deficiency, and elucidate the underlying mechanism of IBS-D and seek out therapeutic targets of TXYF granule based on whole transcriptome analysis.

## Methods/design

### Study design and recruitment

**Fig. 1:**
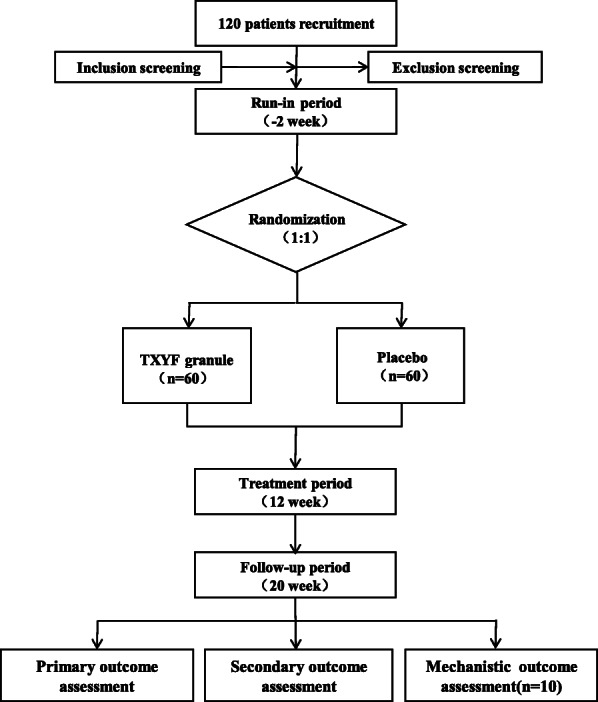
Flowchart of the trial: 120 eligible subjects will be randomly allocated into the TXYF granule group and placebo group in a 1:1 ratio, from which 10 participants of each group randomly will be selected to undergo mechanistic assessment. Colonic samples will be obtained from each group of 10 participants (pre- and post-treatment) and 10 healthy controls for RNA-seq and whole transcriptome analysis

**Fig. 2:**
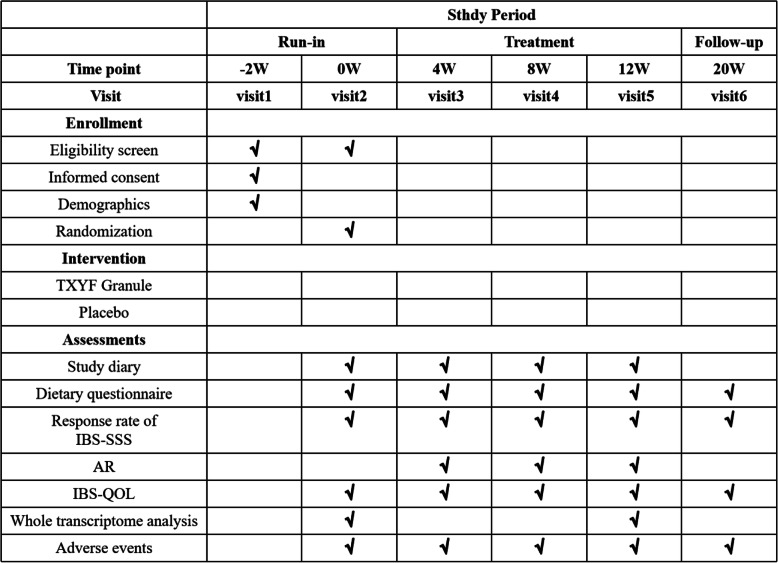
Recommended protocol items: visit schedule for enrollment, interventions, and assessments

This clinical study is a single-center, randomized, double-blind, and placebo-controlled trial comparing TXYF granule with placebo in patients with IBS-D, which will be performed lasting totally 22 weeks. At the initial run-in period (weeks − 2–0), we will assess eligibility and seek informed consent. Then, coming to the treatment period (weeks 0–12), eligible subjects will be randomly assigned to one of two groups (TXYF granule group and placebo group) in a 1:1 ratio and get blinded treatment of 12 weeks. Finally, we will have a follow-up (week 20) 2 months after the treatment. This study involves 6 site visits (week − 2, week 0, week 4, week 8, week 12, and week 20) and 12 call visits (weekly call follow-up during the treatment period); eventually, the primary outcome and the secondary outcome will be evaluated. We will obtain the intestinal mucosae from partial IBS-D patients (*n* = 10 for each group) before and after treatment for the assessment of mechanistic outcome. The process of the study and visit schedule are schematically shown in Figs. 1 and 2. This clinical trial protocol refers to The Standard Protocol Items: Recommendations for Interventional Trials (SPIRIT) 2013 Checklist detailed in Additional file 1.

All 120 IBS-D patients participating in this study will be recruited from the gastroenterology department or ward at the First Affiliated Hospital of Guangzhou University of Chinese Medicine. And we will additionally recruit 10 healthy controls who will undergo intestinal mucosal biopsy for investigation of transcriptome profiling. Written informed consent which contains additional provisions for collection and use of colon samples will be obtained from each participant before enrollment. The trial process conforms to the Declaration of Helsinki (2008) [24]. The Clinical Trials and Biomedical Ethics Committee of the First Affiliated Hospital of Guangzhou University of Chinese Medicine (No.ZYYECK YJ [2018]125) has approved our trial.

### Eligibility criteria

#### Inclusion criteria

1. Patients have to satisfy the diagnostic criteria of IBS-D defined by ROME IV [25].
2. The score of IBS-Symptom Severity Score (IBS-SSS) is more than 75 at baseline visit [26].
3. Patients have to satisfy the diagnostic criteria of diarrhea syndrome with liver depression and spleen deficiency based on TCM syndrome differentiation [27].
4. The age is between 18 and 70 years old.
5. Patients must make an agreement to participate and sign a written informed consent.

#### Exclusion criteria

1. Patients have a history or current evidence of inflammatory bowel disease, gastrointestinal bleeding, or abdominal surgery.
2. Patients have a history or current evidence of other organ diseases which influence gastrointestinal motility, such as hyperthyroidism, diabetes, and chronic renal impairment.
3. Patients have a history or current evidence of serious damages of the heart, liver, kidney, blood system, or immune system.
4. Patients used therapeutic medications for IBS-D or other diseases which could affect the trial within 2 weeks before enrollment.
5. Patients are currently pregnant and lactating.
6. Patients have an allergy to trial medication.
7. Patients have a history of neurological disease or mental illness.
8. Patients have participated in other clinical studies.

### TCM syndrome differentiation

TXYF is typically applied to IBS-D patients with liver depression and spleen deficiency according to the expert consensus on diagnosis and treatment of irritable bowel syndrome in traditional Chinese medicine (2017) [27]. Patients who have two primary symptoms plus one secondary symptom, integrated with signs of the tongue and pulse, will be differentiated as syndrome of liver depression and spleen deficiency and enrolled:

- Primary symptoms: (1) abdominal pain accompanied by diarrhea and (2) prone to irascible or irritable
- Secondary symptoms: (1) distending pain in hypochondrium, (2) reduced appetite, and (3) presented with fatigue
- Auxiliary symptoms: light and enlarged tongue or tooth-marked tongue with white greasy coating, string pulse

### Randomization and blinding

Randomization scheme that generates a list of 120-case randomization sequence utilizing the stratified block randomization method by computer will be implemented by an independent statistician, which guarantees that enrolled patients will be evenly allocated to the TXYF granule group or the placebo group. Then, 10 participants of each group will be randomly selected for RNA sequencing and whole transcriptome analysis. Concealment of allocation code relies on an opaque envelope. This trial requires that all participants and researchers involved in drug distribution, outcome evaluation, and data analysis are completely blinded to allocation. The occurrence of severe adverse events or other unpredictable events allows unblinding under the permission of the principal investigator.

### Experimental medication preparation and quality control

TXYF granule and placebo are uniformly manufactured by Guangdong YiFang pharmaceutical co., LTD under the guidance of Good Manufacturing Practices (GMP), which have the identical appearance and nearly similar taste. TXYF granule is made up of *Rhizoma Atractylodis Macrocephalae*, *Paeoniae Radix Alba*, *Citri Reticulatae Pericarpium*, and *Saposhnikoviae Radix* (see Table 1). These four crude herbs are weighed in proportion of the prescription, subsequently washed, and crushed, and the thickness of the slices is 0.3–1.5 cm. *Rhizoma Atractylodis Macrocephalae* and *Paeoniae Radix Alba* are fired while *Citri Reticulatae Pericarpium* and *Saposhnikoviae Radix* are raw for use. According to the optimized implementation scheme for decocting, extracting, and concentrating of the crude herbs, the ointments are prepared with a relative density of 1.07 to 1.09 g/ml, and then the ointments are screened to get granulated ointments. Finally, after spray drying and crushing, the granules are packed in sealed opaque packages. Placebo consisting of maltodextrin, dextrin, experimental medication extract (< 5%), kudingcha extract, and pigment is also prepared granules. The specific content is shown in Table 2.

**Table 1:** Components of TXYF granule

Chinese name	Botanical name	Latin name	Part used	Source	Dosage (g)
Baizhu	*Atratylodes macrocephala macrocephala* Koidz.	*Rhizoma Atractylodis Macrocephalae*	Rhizome	Zhejiang	18
Baishao	*Paeonia lactiflora* Pall.	*Paeoniae Radix Alba*	Root	Sichuan	12
Chenpi	*Citrus reticulata* Blanco	*Citri Reticulatae Pericarpium*	Matured pericarp	Guangdong	9
Fangfeng	*Saposhnikovia divaricata* (Turez.) Schischk.	*Saposhnikoviae Radix*	Root	Neimeng	6

**Table 2:** Components of the placebo

Ingredients	Component (%)
Maltodextrin	23.97
Dextrin	18.6
Starch	50.0
Experimental medication extract	5.0
Kudingcha extract	2.0
Pigment	0.43

The quality inspection department of Guangdong YiFang pharmaceutical company was responsible for the quality control of experimental medication based on pharmacopeia and national drug quality standards. Thin-layer chromatography and high-performance liquid chromatography-mass spectrometry were respectively utilized for qualitative and quantitative testing. Among them, the content of paeoniflorin in extract of *Paeoniae Radix Alba*, the content of hesperidin in *Citri Reticulatae Pericarpium* extract, and the total content of Prim-O-Glucnylcimifugin and 4′-O-β-glucosyl-5-O-Methylvismmiside in *Saposhnikoviae Radix* extract were 8.97%, 0.84%, and 0.7%, respectively. The microbial limit of each herb extract was qualified.

### Intervention

TXYF granule or placebo will be orally administered one packet (strength, 8.1 g) after dissolved twice a day (1 h after the meal) for 12 weeks. Over the course of the study, participants are required to discontinue therapeutic medications associated with IBS-D. Simultaneously, probiotic foods, functional foods, and dietary supplements are also prohibited. Participants are obligated to fill in the study diary daily involving medication administration record, concomitant treatments, and dietary questionnaire which avail to check compliance and statistically rectify dietary bias. Onset of exacerbated IBS-D symptoms allows rescue medications for less than 1 week, which needs to be documented in the electronic Case Report Form (eCRF).

### Colonoscopy and biopsies

Participants who did not have a colonoscopy 1 year prior to enrollment will undergo colonoscopy and sigmoid mucosal biopsy (week 0) for assessment of microscopic colitis, from which 10 participants of each group will be randomly selected to undergo mechanistic assessment and 2 additional samples of sigmoid mucosa will be obtained. After 12 weeks of treatment, these 20 participants will undergo a second colonoscopy and two sigmoid colon biopsies (week 12). Besides, 10 healthy subjects will be recruited to undergo colonoscopy and two sigmoid colon biopsies. The obtained samples will be promptly snap-frozen in liquid nitrogen and then stored at − 80 °C for the next-generation high-throughput RNA sequencing (RNA-seq) and quantitative real-time PCR (qRT-PCR). Samples of intestinal mucosa will be destroyed after use.

### RNA isolation, library preparation, and sequencing

According to the manufacturer’s instructions, total RNA will be isolated from sigmoid colon samples using TRIzol (Life Technologies). RNA purity, concentration, and integrity by using the NanoPhotometer spectrophotometer (IMPLEN, CA, USA), Qubit RNA Assay Kit, and RNA Nano 6000 Assay Kit respectively will be measured. Two to 3 μg total RNA per sample as input material is needed for cDNA library construction and subsequent PCR amplification and library quality will be evaluated lastly on the Agilent Bioanalyzer 2100 system. RNA-seq will be conducted on an Illumina Hiseq 2000 platform.

### RNA-seq data processing and bioinformatics analysis

Raw data of fastq format through quality control including removing a series of hybrid reads and low-quality reads will be filtered as clean data. Clean data then will be mapped to the human reference genome employing HISAT2 v2.0.4 and the aligned reads per sample will be assembled by StringTie (v1.3.3). Both ncRNAs and coding transcripts will be normalized and expressed as fragments per kilo-base of exon per million fragments mapped (FPKM). Differential expression will be analyzed by using Cuffdiff. Gene Ontology (GO) and Kyoto Encyclopedia of Genes and Genomes (KEGG) enrichment analysis of differentially expressed genes will be implemented by GOseq R package and KOBAS software, respectively. To predict the targets of ncRNAs, databases including TargetScan (www.targetscan.org), starbase (http://starbase.sysu.edu.cn/), deepbase2.0 (http://deepbase.sysu.edu.cn/), and circInteractome (https://circinteractome.nia.nih.gov) will be used. Visual networks of ncRNAs and mRNAs will be constructed by Cytoscape software.

### Outcome measurements

#### Primary outcome

The primary outcome of our trial is the response rate of IBS-Symptom Severity Score (IBS-SSS). A responder is defined as a 50% or more reduction in IBS symptoms compared to baseline [28]. IBS-SSS is a hitherto unique symptom severity questionnaire of IBS, which evaluates the condition of abdominal pain, abdominal distension, and defecation satisfaction. This definition of a responder that reports a 50% or more reduction in IBS symptoms is less disturbed by the initial severity of IBS. IBS-SSS will be assessed at baseline, week 4, week 8, week 12, and week 20 while the response rate of IBS-SSS will be assessed at week 4, week 8, week 12, and week 20.

#### Secondary outcome

##### Adequate relief (AR)

Participants will receive 12 call visits (weeks 1–12) and the following question will be asked: “In the past week, have you had adequate relief of your irritable bowel syndrome pain and discomfort?”. Participants only need to answer “yes” or “no.” Responder is defined as a “yes” answer to the aforementioned question for at least 6 of the 12 weeks. AR will be accessed at week 12.

##### Irritable Bowel Syndrome-Quality of Life Questionnaire (IBS-QOL)

IBS-QOL reflects the union of the physical and mental well-being covering eight dimensions, which will be assessed at baseline, week 4, week 8, week 12, and week 20.

##### Long-term efficacy assessment

Participants will be followed up for long-term effectiveness 2 months after the treatment period (week 20). IBS-SSS response rate and IBS-QOL will be evaluated.

### Mechanistic outcome

We will analyze the whole transcriptome profiling of intestinal mucosae from the TXYF granule group (*n* = 10) and placebo group (*n* = 10) at baseline and week 12 and healthy subjects (*n* = 10) at baseline. The whole set of transcriptional deviations including mRNA, miRNA, lncRNA, circRNA, and functional networks of ncRNAs and mRNAs in IBS-D will be disclosed, which makes us to seek for the underlying molecular mechanisms and find out specific diagnostic biomarker. Meanwhile, therapeutic targets and pathways about TXYF in IBS-D will be clarified. On an Illumina Hiseq 2000 platform, the preparation of whole-set transcriptome libraries and deep sequencing will be executed by Novogene Bioinformatics Technology Cooperation (Beijing, China).

### Safety outcome

The participant will receive relevant laboratory test at baseline and week 12, including liver and kidney function (ALT, AST, Scr, BUN), blood routine test, urine routine test, and fecal occult blood test. The other tests include electrocardiograph examination.

### Adverse events

Adverse events (AEs) defined as any unpredictable and unexpected nocuous effects resulting from TXYF granule or placebo granule throughout the trial will be recorded in detail by a research assistant, including symptom, sign, severity, start date, duration, laboratory result, intervention, and outcome at every visit. Once severe adverse events (SAEs) occur, it must be immediately reported to the principal investigator, the ethics committee of the hospital, and the Guangdong Food and Drug Administration within 24 h and subject safety should be the priority. If necessary, the principal investigator has the right to terminate this trial.

### Sample size calculation

The sample size calculation of this superiority trial depends on our previous pilot study and literature [9]. It can be speculated that the response rates of TXYF granule and placebo will reach 70% and 40%, respectively. We calculate by the software Gpower3.1 under the setting of 80% power and 5% type I error that 42 cases of each group are needed. With a drop-out rate of 15%, accumulated 100 cases are needed and we expect to recruit 120 IBS-D patients.

### Data management and monitor

Data management relies on a convenient follow-up system maintained by Empower Electronic Data Collection (EDC) (Solutions, Shanghai, China), and the company is also responsible for converting paper study diary and any other paper source documents (dietary questionnaire) to the electronic version. The collected data from participants will be kept absolutely secret, and the source data will be just accessible to the data administrator and statistician. All the paper source documentation about this trial will be properly preserved 5 years after the end of the trial.

Our group made up of a principal investigator, 3–5 clinicians, 2 research assistants, a data administrator, a drug manager, and a statistician will receive rigorous training and perform this trial in accordance with the Standard Operating Procedures (SOP) and Good Clinical Practice (GCP). This trial is unconditionally subject to the supervision and monitoring of the Science and Technology Department of the First Affiliated Hospital of Guangzhou University of Chinese Medicine. This independent department is responsible for the annual audit of our trial.

### Statistical analysis

#### Clinical data analysis

Based on the principle of intention-to-treat (ITT), all randomly assigned participants will be included in the statistical analysis. For participants who prematurely terminate this trial, outcomes of the last visit will be used as the final outcomes. Statistical analyses will be completed by SAS (version 9.4, SAS Institute, Cary, NC). Continuous variables will be presented in the form of means and standard deviations while the categorical variables will be presented in the form of counts and percentages. The primary outcome (week 4, week 8, week 12, and week 20) and the secondary outcome AR (week 12) will be assessed by using *χ*^2^ test or Fisher’s exact test. Another secondary outcome IBS-QOL (baseline, week 4, week 8, week 12, and week 20) will be assessed by using *T* test or Mann-Whitney *U* test accompanied with the Wilcoxon test. A two-sided *P* < 0.05 indicates statistical significance. For missing data, sensitivity analysis will be conducted and the optimal approach to the imputation of missing data will be proposed.

#### Mechanistic data analysis

The differential expression analysis about mRNA, miRNA, lncRNA, and circRNA for all pairwise comparisons: IBS-D patients versus healthy controls, TXYF granule (pre-treatment) versus placebo (pre-treatment), TXYF granule (post-treatment) versus placebo (post-treatment), TXYF granule (pre-treatment) versus TXYF granule (post-treatment), and placebo (post-treatment) versus placebo (post-treatment), will be performed by Cuffdiff. An adjusted *P* < 0.05 (Student’s *t* test accompanied with Benjamini-Hochberg FDR adjustment) will be used as the cut-off for significantly differentially expressed genes. Pearson correlation test will be used for target gene prediction.

## Discussion

Previous meta-analysis [29] and another two clinical trials [9, 30] have demonstrated with adequate evidence that TXYF or Tongxie formula (modified TXYF) is pretty effective and safe compared with conventional medications or placebo. Disease and syndrome differentiation are the crux of TCM treatment as we know, TXYF accords with one kind of principal pathogenesis known as liver depression and spleen deficiency of IBS-D in TCM theory so it can work. Some clinical trials with TXYF did not limit the syndrome type, which could reduce statistical power and increase type 2 errors. And in previous trials, high heterogeneity in composition and manufacture of TXYF biases the clinical outcomes and also confuses mechanistic results. The design of our trial complies with rigorous methodology and quality control. TXYF granule is produced with the standardized procedure, which guarantees homogeneous active components and makes outcomes more credible. Then, there is the issue of course of treatment. Long-term follow-up reports about this kind of functional bowel disease are rare because IBS-D has the clinical feature of recurrent episodes. IBS-D has many triggers, of which diet and emotion stress show high inducibility of this disease [31, 32]. So, we prolong the treatment period to 12 weeks in order to reduce symptom relapse and obtain better long-term efficacy. This naturally brings the problem of patient compliance, which requires us more attention.

This trial not only explores the efficacy and safety of TXYF granule but also focuses on the whole transcriptome profiling of IBS-D and the therapeutic pathways of TXYF granule. A previous study has concluded that miRNAs play a pivotal role in the IBS rat model by utilizing liquid chip and qRT-PCR and might be the targets of TXYF in the treatment of IBS [33]. Other literatures have reported that in IBS-D patients, miR-199 and miR-29 are respectively involved in visceral pain and intestinal permeability [34, 35]. Our previous studies also have proved that miR-144 and miR-200 by binding to specific target mRNA play vital roles in IBS-D rats [22, 23]. However, the study on lncRNAs and circRNAs in IBS-D is as yet scarce. Although IBS-D belongs to one kind of functional bowel disease, the mucosal functions resulting from the abnormality of relevant expressed genes and gene-coding proteins have a confirmed discrepancy between IBS-D patients and healthy individuals [36]. Especially for recognized mechanisms of IBS-D including visceral sensitivity, intestinal permeability, and intestinal motility, we anticipate starting with the target genes involved the three aspects to seek for the upstream regulatory mechanism of coding and noncoding RNAs. It is precisely because of the multiplicity of mechanism in IBS-D and pleiotropic effects about herbal treatment that genetic and transcriptional level studies can provide a penetrating and comprehensive perspective on the molecular mechanism of IBS-D and therapeutic targets of TXYF. This will be the first study to probe the whole transcriptome profiling of IBS-D and also will be the first study to explore the therapeutic targets or pathways of TCM employing whole transcriptome analysis in IBS-D.

Limitations still exist in our study. Firstly, patients who underwent the first colonoscopy and biopsies may not want to have the second colonoscopy and biopsies after the treatment, especially for those with obvious remission of symptoms. We will fully communicate with them and give careful explanation and emphasize this point before the enrollment. Secondly, because of the long period of treatment and follow-up, we will encounter the problem of high drop-out rate and establishing close contact with participants is a must. In conclusion, we hope this trial will provide high-quality evidence on the effectiveness and safety of TXYF granule. We also expect to have more insight into the mechanism of IBS-D and find out specific biomarkers and pinpoint the targets of TXYF in IBS-D.

## Trial status

This is protocol version 3 dated 15 June 2019. We made further amendment on the basis of version 1 dated 30 September 2018, and we will communicate the protocol amendments to the Clinical Trials and Biomedical Ethics Committee of the First Affiliated Hospital of Guangzhou University of Chinese Medicine. Because of the COVID-19 pandemic, recruitment will delay to June 2020, and it is anticipated that this trial will be completed in December 2021.

## Supplementary information

**Additional file 1.** SPIRIT 2013 Checklist: Recommended items to address in a clinical trial protocol and related documents.

## Data Availability

The datasets supporting the findings of our study are available from the corresponding author upon request.

## Abbreviations

IBS: Irritable bowel syndrome
TCM: Traditional Chinese medicine
IBS-D: Diarrhea-predominant irritable bowel syndrome
BM: Bowel movements
TXYF: Tong-Xie-Yao-Fang
ncRNAs: Noncoding RNAs
IBS-SSS: IBS-Symptom Severity Score
AR: Adequate relief
IBS-QOL: IBS-Quality of Life Questionnaire IBS-QOL
GMP: Good Manufacturing Practices
eCRF: Electronic Case Report Form
RNA-seq: RNA sequencing
qRT-PCR: Quantitative real-time PCR
FPKM: Fragments per kilo-base of exon per million fragments mapped
GO: Gene Ontology
KEGG: Kyoto Encyclopedia of Genes and Genomes
AEs: Adverse events
SAEs: Severe adverse events
EDC: Electronic data collection
SOP: Standard Operating Procedures
ITT: Intention-to-treat
